# Hippo Signaling Mediates TGFβ-Dependent Transcriptional Inputs in Cardiac Cushion Mesenchymal Cells to Regulate Extracellular Matrix Remodeling

**DOI:** 10.3390/jcdd10120483

**Published:** 2023-12-04

**Authors:** Mrinmay Chakrabarti, Ahad Chattha, Abhijith Nair, Kai Jiao, Jay D. Potts, Lianming Wang, Scotty Branch, Shea Harrelson, Saeed Khan, Mohamad Azhar

**Affiliations:** 1 Department of Cell Biology and Anatomy, School of Medicine, University of South Carolina, Columbia, SC 29202, USA; mrinmay.chakrabarti@uscmed.sc.edu (M.C.); achattha@email.sc.edu (A.C.); jnjithunair@gmail.com (A.N.); jay.potts@uscmed.sc.edu (J.D.P.); 2 Center for Biotechnology & Genomic Medicine, Medical College of Georgia, Augusta University, Augusta, GA 30912, USA; kaijiao@augusta.edu; 3 Department of Statistics, University of South Carolina, Columbia, SC 29208, USA; wangl@stat.sc.edu; 4 KOR Life Sciences, KOR Medical, and Vikor Scientific, Charleston, SC 29403, USA; sbranch@vikorscientific.com (S.B.); sharrelson@vikorscientific.com (S.H.); skhan@vikorscientific.com (S.K.); 5 William Jennings Bryan Dorn VA Medical Center, Columbia, SC 29202, USA

**Keywords:** transforming growth factor beta, Hippo, cushion mesenchyme, heart valve, extracellular matrix

## Abstract

The transforming growth factor beta (TGFβ) and Hippo signaling pathways are evolutionarily conserved pathways that play a critical role in cardiac fibroblasts during embryonic development, tissue repair, and fibrosis. TGFβ signaling and Hippo signaling are also important for cardiac cushion remodeling and septation during embryonic development. Loss of TGFβ2 in mice causes cardiac cushion remodeling defects resulting in congenital heart disease. In this study, we used in vitro molecular and pharmacologic approaches in the cushion mesenchymal cell line (tsA58-AVM) and investigated if the Hippo pathway acts as a mediator of TGFβ2 signaling. Immunofluorescence staining showed that TGFβ2 induced nuclear translocation of activated SMAD3 in the cushion mesenchymal cells. In addition, the results indicate increased nuclear localization of Yes-associated protein 1 (YAP1) following a similar treatment of TGFβ2. In collagen lattice formation assays, the TGFβ2 treatment of cushion cells resulted in an enhanced collagen contraction compared to the untreated cushion cells. Interestingly, verteporfin, a YAP1 inhibitor, significantly blocked the ability of cushion cells to contract collagen gel in the absence or presence of exogenously added TGFβ2. To confirm the molecular mechanisms of the verteporfin-induced inhibition of TGFβ2-dependent extracellular matrix (ECM) reorganization, we performed a gene expression analysis of key mesenchymal genes involved in ECM remodeling in heart development and disease. Our results confirm that verteporfin significantly decreased the expression of α-smooth muscle actin (*Acta2*), collagen 1a1 (*Col1a1*), *Ccn1* (i.e., *Cyr61*), and *Ccn2* (i.e., *Ctgf*). Western blot analysis indicated that verteporfin treatment significantly blocked the TGFβ2-induced activation of SMAD2/3 in cushion mesenchymal cells. Collectively, these results indicate that TGFβ2 regulation of cushion mesenchymal cell behavior and ECM remodeling is mediated by YAP1. Thus, the TGFβ2 and Hippo pathway integration represents an important step in understanding the etiology of congenital heart disease.

## 1. Introduction

Defective heart valve formation is a major cause of congenital heart defects (CHDs) [1]. A bicuspid aortic valve (BAV) is found in up to 2% of the general population [2,3,4]. Atrioventricular (AV) septal defects (AVSDs) comprise 5–10% of all CHDs and are defined as a variable degree of the atrial and ventricular septal defect along with a common or partially separate atrioventricular (AV) orifice [5,6]. Cardiac outflow tract (OFT) defects and abnormalities are estimated to cause approximately 30% of all CHDs [7]. Normal heart development requires that cells integrate signals from various signaling pathways, such as the Hippo and transforming growth factor-beta (TGFβ), which are major regulators of tissue growth, apoptosis, organ size, and differentiation [8,9,10]. Cell–cell contact inhibition and growth can be regulated by TGFβ but in a Hippo-independent manner [11]. These pathways are intricately involved in cardiac fibroblasts during heart development and postnatal cardiac tissue repair and cardiac fibrosis [9]. There are three TGFβ ligands in mammals [12,13]. Genetic mutations in TGFB2 are found in many syndromic patients with a connective tissue disorder with CHD caused by abnormal AV and OFT cushion remodeling resulting in aortic and/or mitral valve malformations [14,15]. All TGFβ ligands bind to the heteromeric TGFβ receptor complex leading to the phosphorylation of canonical SMAD2 or SMAD3. Phosphorylated SMAD2/3 function as activated SMADs, and in conjunction with SMAD4, they translocate to the nucleus. The three multifunctional TGFβ cytokines, which upon interaction with its receptors induces both “canonical”, i.e., SMAD2/3-dependent, and noncanonical, i.e., non-SMAD (p38 and ERK1/2 MAPK-mediated) signaling, are the main inducers of EMT and fibrogenesis [16]. An activated SMAD2/3/4 complex binds to TGFβ response elements in the promoter of target genes and regulated transcription. TGFβ ligands are profibrotic molecules which regulate key genes involved in extracellular matrix (ECM) remodeling and (myo)fibroblast activation, including α-SM actin (Acta2), Collagen 1a1 (Col1a1), and Cellular Communication Network Factor 2 (*Ccn2*) (also known as CTGF) [13,17]. The transcriptional regulatory mechanisms by which specific TGFβ ligands regulate ECM remodeling in cardiac cushion remodeling have not been fully resolved.

The Hippo signaling pathway modulates cardiac fibroblast activation and regenerative response by regulating cardiomyocyte and fibroblast proliferation and ECM remodeling in cardiac development and cardiomyopathy [9,18,19]. The Hippo signaling mechanisms were first investigated in *Drosophila*. The Hippo signaling pathway is crucial for the development of several organs, including the heart, by controlling cell proliferation, apoptosis, cell fate decisions, and stem cell self-renewal [20]. The mammalian core components of the Hippo signaling pathway are composed of a kinase cascade, several transcription factors, and coactivators, like Mst1/2, *Sav1*, Lats1/2, Mob1, and Yap/Taz [20,21]. Activation of the protein cascade leads to phosphorylation, cytoplasmic retention, and degradation of the transcriptional coactivators, Yes-associated protein 1 (YAP1) and transcriptional coactivator with PDZ-binding motif (TAZ) [20]. In contrast, inactivation of upstream kinases resulted in nuclear translocation of both YAP1 and TAZ, where they further interact with various transcription factors, including SMAD family members [8]. This results in regulation of transcriptional activity for downstream target genes involved in cell proliferation, survival, differentiation, and ECM remodeling [9,18,20,21]. Cellular Communication Network Factor 1 (CCN1/CYR61) and CCN2 (also known as connective tissue growth factor, CTGF) are among major targets of the Hippo pathway involved in ECM remodeling, cell proliferation, and cell survival [20].

Hippo/YAP are required for epithelial–mesenchymal transition (EMT) during heart cushion formation at E9.5. YAP deletion in cushion endothelial cells reduced endocardial cell proliferation, resulting in hypoplastic cardiac cushions and increased embryonic lethality [22]. YAP is widely expressed in post-EMT valves at E15.5, suggesting that Hippo/YAP also play a role in post-EMT cushion remodeling and valve morphogenesis. VGLL4, a transcriptional corepressor in the Hippo pathway, is critical for balancing cell proliferation and apoptosis during development and tissue homeostasis. VGLL4 and YAP do not contain a DNA-binding domain, and they mediate their biological functions mainly through interaction with the TEAD transcription factor family [23]. It has been reported that endothelial but not neural crest VGLL4 regulates valve interstitial cell proliferation (VIC). This could be due to the fact that VGLL4 is mainly expressed in valve endothelial cells (VECs) and endothelial-derived VIC. Thus, Hippo signaling in VEC is required for post-EMT semilunar valve morphogenesis and homeostasis. Collectively, the role of Hippo signaling in valve formation and morphogenesis remains to be fully investigated.

There are many studies indicating that TGFβ-induced SMAD2/3 localization is mediated by YAP and TAZ [8]. The activity of YAP and TAZ is determined by their localization within the cell, with nuclear protein being ‘active’ and cytosolic protein being ‘inactive’. Furthermore, treating cells and mice with verteporfin, a well-characterized YAP1 inhibitor [24], blocks TGFβ-induced myofibroblast activation.

ECM remodeling represents a key step in cardiac cushion remodeling and maturation and cardiac septation [25]. Given the role of the TGFβ and Hippo pathways in fibrogenesis and ECM remodeling, it is plausible that these pathways integrate and control ECM remodeling in heart development. It is thought that there are retention factors in the nucleus that have a higher affinity for phosphorylated SMADs [26]. However, the regulation of SMAD nuclear accumulation, which governs the transcription of target genes, is still not very well understood [27]. Thus, to understand signaling cross-talk between the TGFβ–SMAD and YAP1 signaling pathways in cardiac cushion remodeling in heart development, we investigated the role of YAP1 in SMAD nuclear accumulation in immortalized embryonic cushion mesenchymal cells. We used cushion mesenchymal cell cultures and molecular and biochemical approaches to determine if Hippo signaling via YAP1 is essential for TGFβ2-dependent cushion mesenchymal cell-matrix remodeling. Collectively, our results confirm that the YAP1-dependent pathway regulates the TGFβ-induced transcriptional regulatory mechanisms involved in ECM remodeling in cardiac cushion mesenchyme.

## 2. Material and Methods

### 2.1. Cushion Mesenchymal Cell Culture and Drug Treatment

The tsA58-AVM cell line was derived from AV cushions of H-2Kb-tsA58 embryos at E9.5 [28]. In H-2Kb-tsA58 animals, expression of the temperature-sensitive SV40 large T antigen mutant gene (*tsA58*) is driven by the γ-interferon-inducible promoter of the *H-2Kb* gene. Under the permissive condition (33 °C, with γ-interferon), cushion mesenchymal cells proliferate continuously like immortalized cells. However, under the restrictive condition (37 °C, no γ-interferon), the large T antigen is degraded, and these cells cease to proliferate and resemble primary cell cultures. Thus, cushion mesenchymal cells were grown at 33 °C in DMEM media containing 10% FBS, 1% penicillin–streptomycin, glutamine (1 mM), and γ-interferon (10 units/mL; Peprotechn INC. Cranbury NJ, USA; catalog: 315-05). Once cells were ~80–90% confluent, they were seeded in 100 mm dishes or well plates for drug treatments at 37 °C without γ-interferon. At the end of the treatments, cells were washed thrice in 1xPBS and collected for different experiments.

### 2.2. Immunofluorescence Staining and Immunoblotting

Cushion mesenchymal cells were cultured on the 2-well BD chamber slides as discussed above. When 80–90% confluent, cells were starved with 0.5% FBS/DMEM media for 24 h and then treated with TGFβ2 (5 ng/mL, R&D Systems, Inc., Minneapolis, MN, USA) for 12 h. A set of untreated controls was also kept to compare with the TGFβ2-treated cells. Cells were then washed thrice with PBS (1×) and stained with antibodies directed against SMAD3 or YAP1 both at a dilution of 1:500 (Table 1). Primary IgG antibodies of SMAD3 and YAP1 were purchased from Cell Signaling Technology, Inc. (Danvers, MA, USA) and abcam (Cambridge, MA, USA), respectively. The Cy3 goat anti-rabbit secondary IgG antibody (ThermoFisher Scientific, Grand Island, NY, USA) was used at 1µg/mL dilutions to detect the primary IgG antibody. After the addition of secondary antibodies, nuclear counterstaining with DAPI was performed. Immunofluorescence microscopic images were obtained using Leica Thunder imager (Leica Microsystems Inc., Buffalo Grove, IL, USA), and the relative intensities of SMAD3 or YAP1 immunofluorescence were calculated using ImageJ2 software (National Institutes of Health, Bethesda, MD, USA). We measured 7–8 fields of different images using ImageJ software. The data were analyzed and graphically presented using GraphPad Prism 9.0 software (San Diego, CA, USA). All experiments were performed at least three times. The results are presented as bar graph and dots representing individual data points.

### 2.3. Collagen Gel Contraction Assays

Cushion mesenchymal cells were maintained in DMEM (Invitrogen, Waltham MA, USA) supplemented with 10% bovine serum, 5% fetal calf serum, and 1% penicillin/streptomycin (Sigma-Aldrich, St. Louis, MO, USA) at 33 °C/5% CO_2_ as discussed above. In addition to reorganization, collagen contraction assays are indicative of the capacity for embedded cells to generate mechanical loads. The capability of cushion cells to form lattices in collagen gels was assessed by plating 10^5^ cells in 2 mg/mL collagen type-I (in 18 mM acetic acid) prepared in complete media and supplemented with 0.1 M NaOH, as detailed in [29,30]. Free-floating collagen gels were incubated at 37 °C for 5 days, with or without recombinant TGFβ2 (5 ng/mL, R&D Systems, Inc., Minneapolis, MN, USA) and verteporfin (5 µg/mL, Tocris/Bio-Techne, Minneapolis, MN, USA). Images were acquired, and best-fit shapes were used to assess the number of pixels to calculate the % area or diameter of the collagen gels using ImageJ software. The relative change in % area or diameter (AU) of gels after 5 days denotes the degree of collagen contraction, with lower values indicating greater contraction [29,31]. The results are presented as bar graph and dots representing individual data points.

### 2.4. RNA Isolation, cDNA Synthesis, and Quantitative PCR

mRNA was isolated using Trizol (Invitrogen, Waltham MA, USA) and miRNeasy micro kit (Qiagen, Germantown, MD, USA) according to manufacturer’s protocols, and cDNA was generated from 1 µg mRNA using Invitrogen kit according to manufacturer’s instructions. A sample of 20 ng cDNA was subjected to quantitative PCR amplification (Biorad-CFX) using pre-validated gene-specific primers procured from the vendor (Biorad Inc., Hercules, CA, USA). Following PCR analyses, the cycle count threshold (Ct) was normalized to species-specific housekeeping genes and β2-microglobulin (*B2M*; purchased from Biorad Inc.), and the ∆Ct and fold changes in experimental samples over controls were determined (Table 2). Statistically significant differences in gene expression levels were determined using unpaired one-tailed Student’s *t*-test and are indicated in the figures, in at least 3 independent experiments with *p* < 0.05 considered significant.

### 2.5. Western Blot Analysis

Western blotting was performed with the protein extracted from the cell lysate of cushion mesenchymal cells following different treatments [29,31]. The cells were homogenized using Wheaton tapered tissue grinders (Thermo Scientific, Rockford, IL, USA) in M-PER mammalian protein extraction reagent (Thermo Scientific, Rockford, IL, USA) with complete mini protease inhibitor cocktail (Sigma-Aldrich, St. Louis, MO, USA) and Halt protease and phosphatase inhibitor single-use cocktail (ThermoScientific, Rockford, IL, USA) as per the manufacturer’s protocol. Homogenized tissue lysates were subjected to brief sonication for 20 s on ice and kept at room temperature for 20 min. Then, centrifugation was performed at 15,000 rpm for 20 min at 4 °C and the supernatants were collected. Total protein concentration in the supernatant was determined using Pierce BCA protein assay kit (Thermo Scientific, Rockford, IL, USA), and the samples were stored at −80 °C until further use. Western blotting was performed with equal amounts of protein samples and the primary IgG antibodies against phospho-SMAD2 (Cell Signaling Technology, Danvers, MA, USA; cat #3108), SMAD2 (Cell Signaling, Inc.; cat #5339), phospho-SMAD3 (Cell Signaling Inc., cat #9520), SMAD3 (Cell Signaling Inc., cat #9523), and YAP1 (Abcam, Cambridge, MA, USA; cat #ab205270) at a dilution of 1:1000. Primary IgG antibodies against all these proteins were purchased from Cell Signaling Technology, Inc. (Danvers, MA, USA) or Abcam (Cambridge, MA, USA). The horseradish peroxidase-conjugated anti-mouse or anti-rabbit secondary IgG antibody (Cell Signaling, cat#7074) was used at 1:5000 dilutions to detect a primary IgG antibody. In a separate Western blot, the levels of β-actin in all cell lysate samples were determined. Western blots were incubated with Clarity Western ECL detection reagents (Bio-Rad Laboratories, Hercules, CA, USA) and exposed to X-OMAT AR films (Eastman Kodak, Rochester, NY, USA) for autoradiography. The autoradiograms were scanned on an EPSON scanner using Photoshop software v25.2 (Adobe Systems, Seattle, WA, USA). β-actin, clone AC-15 monoclonal primary antibody (Sigma-Aldrich, St. Louis, MO, USA) was used as an internal housekeeping control to compare equal loading in the SDS-PAGE. The ratios of both phosphorylated protein/nonphosphorylated protein or β-actin were plotted as scatter dot plots with the box denoting the mean ± SEM and dots representing individual data points.

### 2.6. Statistical Analysis

Microsoft Excel was used for recording and managing the raw data. Data were presented as mean ± SEM. Continuous data were presented as bar/dot plots, showing the individual data points together with the average/error bars. Statistical significance was calculated using Student’s *t* test (two-tailed, independent two-sample *t*-test) using the GraphPad Prism 8 statistical program (GraphPad, San Diego, CA, USA). We considered pairwise comparisons directly, which revealed whether the considered pairs of population means were different. Exact *p*-values were calculated, and the probability values <0.05 were considered as significant. The *p*-values are indicated in all figures.

## 3. Results

### 3.1. Exogenous TGFβ2 Induced SMAD3 Activation in Cushion Mesenchymal Cells

**Figure 1:**
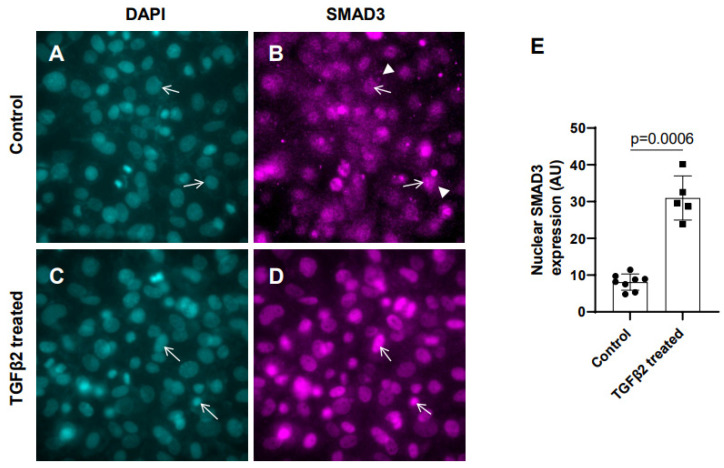
TGFβ2 induces nuclear translocation of SMAD3 in cushion mesenchymal cells. (**A**–**D**) Cushion mesenchymal cells were grown on 2-well chamber slides at 80–90% confluency and were untreated (control) (**A**,**B**) or treated with TGFβ2 (5 ng/mL) for 12 h (**C**,**D**). Cells were stained with primary SMAD3 antibodies, followed by secondary Cy3 goat anti-rabbit IgG (pink color) and counterstained with DAPI (teal color) to identify nuclei. Representative images confirmed enhanced nuclear accumulation of SMAD3 following treatment with TGFβ2 (**D**). The nuclear SMAD3 fluorescence intensity was measured for the untreated control and TGFβ2-treated cells and presented graphically by a scatter plot with bar showing individual values. The error bars indicate mean ± SD. Arrows (**A**–**D**) indicate nuclei, and arrowhead (**B**) denotes cytoplasm. (**E**). Statistically significant differences between control and TGFβ2-treated cells (*p* = 0.0006, two-tailed Student’s *t* test with Welch’s correction; control, *n* = 8: TGFβ2-treated, *n* = 5) were determined and are indicated on the scatter plot.

In the absence of TGFβ2, SMAD2/3 which are not phosphorylated, remain mostly in the cytoplasm. TGFβ stimulation triggers TGFβ receptor-dependent SMAD2/3 phosphorylation at the C-terminal end, which activates SMAD3. Activated forms of both SMAD2/3 then translocate to the nucleus and induce a context-dependent expression of TGFβ target genes to regulate cellular behavior and ECM remodeling. To determine the effect of TGFβ2 on SMAD3 activation, the cushion mesenchymal cells were treated with 1× PBS (control) or TGFβ2 for 12 h. An equal number of cushion mesenchymal cells were used in all independent experiments. Cushion cells were collected, and immunofluorescence staining using anti-SMAD3 antibody was performed. Nuclei were stained. The levels of SMAD3 in the nucleus for the control and TGFβ2-treated cells were quantified. In control samples, SMAD3 predominantly remained in the cytoplasm (Figure 1A,B, arrowheads in B). The data indicate that there was an increased accumulation of SMAD3 in the nucleus (i.e., activated or phosphorylated form of SMAD3 or pSMAD3) of cushion mesenchymal cells following TGFβ2 treatment (Figure 1C,D, arrows). A quantitative analysis confirmed that TGFβ2 treatment significantly upregulated the nuclear SMAD3 (i.e., pSMAD2) in cushion mesenchymal cells compared to control cells (control, *n* = 8; TGFβ2-treated, *n* = 5; *p* = 0.0006) (Figure 1E).

### 3.2. TGFβ2 Treatment Leads to Nuclear Translocation of YAP1 in Cushion Mesenchymal Cells

**Figure 2:**
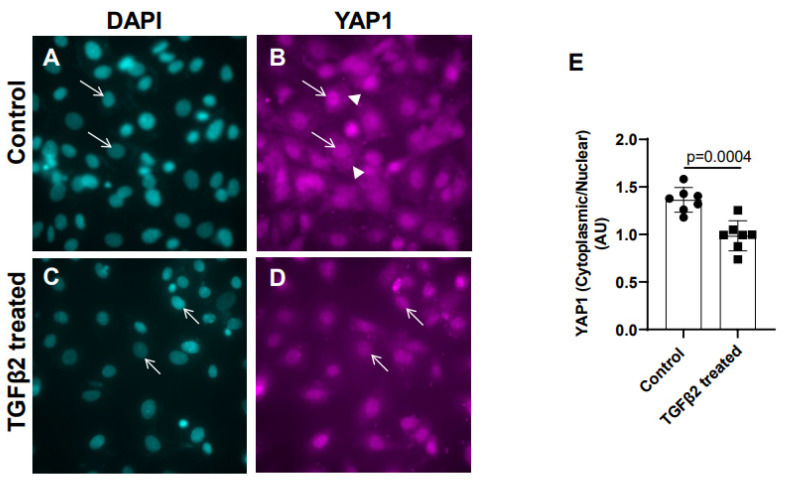
TGFβ2 treatment alters cytoplasmic and nuclear localization of YAP1 protein in cushion mesenchymal cells. (**A**–**D**) Cushion mesenchymal cells were grown on 2-well chamber slides at 80–90% confluency and were untreated (control) (**A**,**B**) and treated with TGFβ2 (5 ng/mL) for 12 h. Cells were stained with primary YAP1 antibodies followed by secondary Cy3 goat anti-rabbit IgG (pink color) and counterstained with DAPI (teal color) to identify nuclei. Representative images confirmed altered cytoplasmic/nuclear localization of YAP1 following treatment with TGFβ2. Arrows (**A**–**D**) indicate nuclei, and arrowhead (**B**) denotes cytoplasm. (**E**) The YAP1 fluorescence intensity in cytoplasm and nucleus was measured in 7–8 fields of different images from the untreated control and TGFβ2-treated cells by using NIH ImageJ software. The ratio of cytoplasmic/nuclear fluorescence intensity was presented graphically by a scatter plot with bar showing individual values. The error bars indicate mean ± SD. Statistically significant differences between control and TGFβ2-treated cells (*p* = 0.0004, two-tailed Student’s *t* test with Welch’s correction; control, *n* = 7; TGFβ2-treated, *n* = 7) were determined and are indicated on the scatter plot.

Cushion mesenchymal cells were cultured and treated with TGFβ2 for 12 h, and immunofluorescence analysis was performed using YAP1 antibody to determine the levels of cytoplasmic and nuclear YAP1. Cytoplasmic YAP1 is predominantly a phosphorylated form of YAP1, whereas nuclear YAP1 is dephosphorylated. The fluorescence signal was quantified, and the ratio of cytoplasmic to nuclear YAP1 levels were compared in both the control and TGFβ2-treated cushion cells. Equal numbers of cushion cells were used in the independent experiments. The data show a higher expression of YAP1 in the cytoplasm of the control cushion cells compared to TGFβ2-treated cushion cells (Figure 2A,B, arrowheads in B). Following TGFβ2 treatment, the levels of cytoplasmic YAP1 were decreased, but most of the YAP1 was translocated into the nucleus (Figure 2C,D, arrows). A quantitative analysis comparing the ratio of cytoplasmic to nuclear YAP1 in the control and TGFβ2-treated cushion mesenchymal cells indicated that the overall ratio of cytoplasmic to nuclear YAP1 was significantly decreased in the TGFβ2-treated cultures compared to the control cushion cells (control, *n* = 7; TGFβ2-treated, *n* = 7; *p* = 0.0004) (Figure 2E).

### 3.3. Suppression of YAP1 Inhibits TGFβ2-Induced Cell-Matrix Remodeling in Cushion Mesenchymal Cells

**Figure 3:**
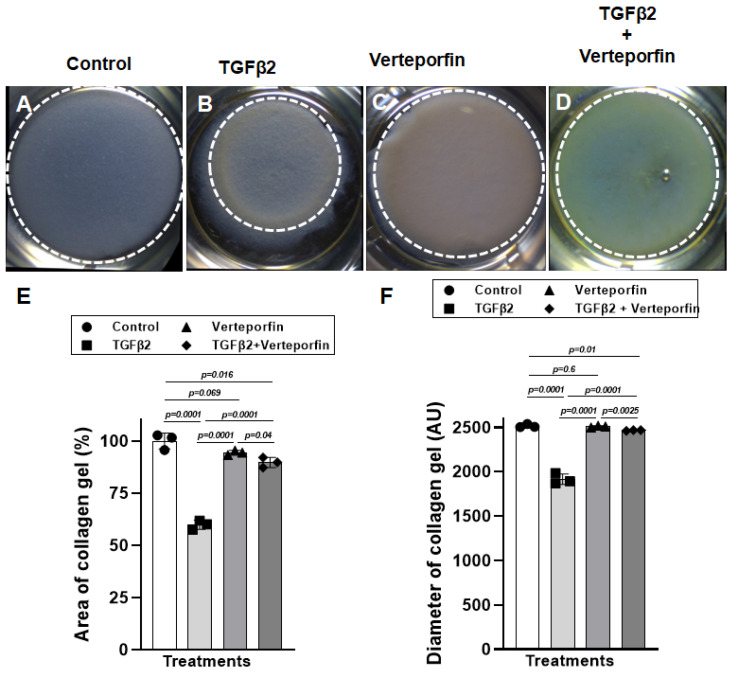
Verteporfin blocks the ability of cushion mesenchymal cells to reorganize and contract collagen gel by exogenously added TGFβ2. (**A**–**D**) Collagen lattice formation assay. Representative images of cushion mesenchymal cells–laden collagen gels 5 days after treatment with TGFβ2 (5 ng/mL) and/or verteporfin (5 µg/mL). TGFβ2-treated cushion cells showed enhanced ability to contract collagen gel (**B**) compared to the untreated cells (**A**). Verteporfin alone had no effect on collagen contraction (**C**), but it significantly blocked the collagen contraction in the presence of TGFβ2 (**D**). (**E**) Collagen gel area (%) (**E**) and diameter (**F**) were measured using NIH ImageJ software, and the numerical data are presented as a scatter plot with bar showing individual values. White dotted circles outlined the collagen gels to reinforce the changes in collagen gel circumference. The error bars indicate mean ± SD. Statistically significant differences of collagen gel diameter between two individual groups (two-tailed Student’s *t* test with Welch’s correction; *n* = 3 each group) were determined, and *p*-values are indicated on the scatter plot.

The cushion mesenchymal cells were cultured in a collagen gel with or without exogenous TGFβ2 stimulation in the presence or absence of verteporfin, a well-characterized inhibitor of YAP1. Cushion cells were grown for 5 days, and TGFβ2 and/or verteporfin was replenished every day. An equal number of cushion mesenchymal cells were used in all independent experiments (*n* = 3). At the end of the experiment, the % area and diameter of the collagen gels were measured for all four treatment groups (control, TGFβ2 treatment, verteporfin treatment, TGFβ2 and verteporfin treatment). The data indicate that the TGFβ2 treatment significantly reduced the collagen gel % area (*n* = 3, *p* = 0.0001) and diameter (*n* = 3, *p* = 0.0001) compared to control (Figure 3A,B,E,F). Verteporfin had no significant effect on % area (*n* = 3, *p* = 0.069) and diameter (*n* = 3, *p* = 0.6) of collagen gels compared to the untreated control (Figure 3A,C,E,F). However, verteporfin significantly blocked the reduction in the collagen gel % area (*n* = 3, *p* = 0.0001) or diameter (*n* = 3, *p* = 0.0001) by cushion mesenchymal cells in the presence of TGFβ2 (Figure 3A–F).

### 3.4. YAP1 Inhibitor Attenuated TGFβ2-Induced Expression of Genes Involved in Cushion Mesenchymal Cell-Matrix Remodeling

**Figure 4:**
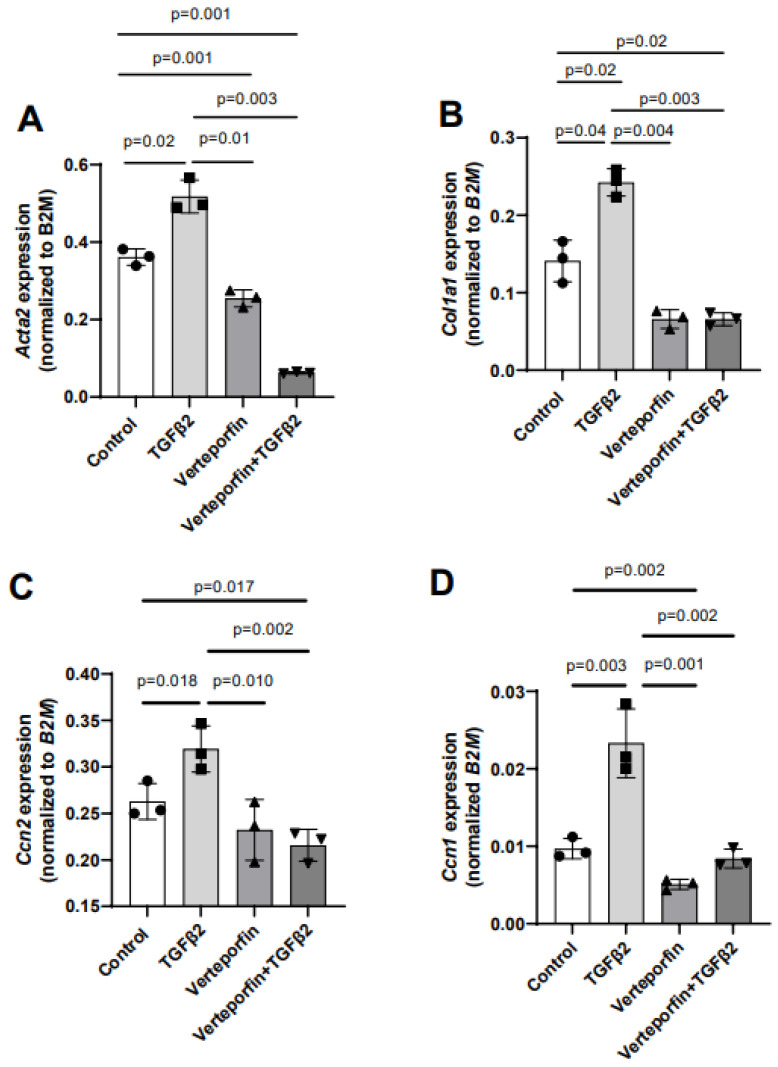
Verteporfin reduces the transcript levels of TGFβ2-induced cushion mesenchymal genes involved in extracellular matrix remodeling. (**A**–**D**) Quantitative real-time PCR analysis showing gene transcript levels of α-smooth muscle actin (Acta2) (**A**), collagen Ia1 (Col1a1) (**B**), connective tissue growth factor/cell communication network 2 (Ctgf/Ccn2) (**C**), and cysteine-rich angiogenic inducer 61/cell communication network 1 (Cyr61/Ccn1) (**D**) in cushion mesenchymal cells in the absence or presence of TGFβ2 (5 ng/mL) and/or verteporfin (5 µg/mL) for 12 h. Gene expression was normalized by B2M, and the numerical data are presented as a scatter plot with bar showing individual values. The error bars indicate mean ± SD. Statistically significant differences of gene expression between two individual groups (one-tailed Student’s t test with unpaired; *n* = 3 each group) were determined, and *p*-values are indicated on the scatter plot.

Given the effect of verteporfin in mediating the TGFβ2-dependent collagen gel organization and contraction, qPCR was used to quantify the alteration in the expression of TGFβ2-induced mesenchymal genes following YAP1 inhibitor (verteporfin) treatment. The cushion mesenchymal cells were treated with TGFβ2 and/or verteporfin for 12 h, and total cellular mRNA was extracted. Quantitative RT-PCR was performed to determine the expression of α-smooth muscle actin (*Acta2*), collagen I (*Col1a1*), connective tissue growth factor/cell communication network factor 2 (*Ctgf* or *Ccn2*), and cysteine-rich angiogenic inducer 61/cell communication network factor 1 (*Cyr61* or *Ccn1*). Equal numbers of cells were used for various treatment and for RNA extraction in all independent experiments (*n* = 3). The data indicate that TGFβ2 significantly induced the expression of *Acta2* (*n* = 3, *p* = 0.02) (Figure 4A), *Col1a1* (*n* = 3, *p* = 0.04) (Figure 4B), *Ccn2* (*n* = 3, *p* = 0.018) (Figure 4C), and *Ccn1* (*n* = 3, *p* = 0.003) (Figure 4D). Verteporfin treatment significantly inhibited the expression of *Acta2*, *Col1a1*, and *Ccn1*. However, the effect of verteporfin on *Ccn2* was not significant (Figure 4A–D). Importantly, verteporfin significantly reduced the TGFβ2-induced upregulation of *Acta2* (*n* = 3, *p* = 0.003) (Figure 4A), *Col1a1* (*n* = 3, *p* = 0.003) (Figure 4B), *Ccn2* (*n* = 3, *p* = 0.002) (Figure 4C), and *Ccn1* (*n* = 3, *p* = 0.002) (Figure 4D). Collectively, the results show that verteporfin effectively decreased the expression of key TGFβ2-induced genes involved in cushion mesenchymal cell-matrix remodeling.

### 3.5. YAP1 Inhibition Decreases Activation of SMAD2 and SMAD3

**Figure 5:**
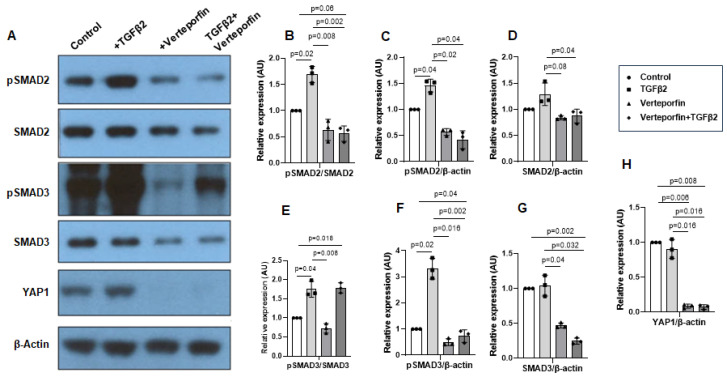
Verteporfin blocks YAP1- and TGFβ2-dependent activation of SMAD2 and SMAD3 in cushion mesenchymal cells. (**A**) Western blot analysis of control and TGFβ2- and/or verteporfin- treated cushion mesenchymal cells showing protein levels of the phosphorylated SMADs (pSMAD2 and pSMAD3), total SMADs (SMAD2 and SMAD3), YAP1, and β-actin. (**B**–**H**) Densitometric quantification of pSMAD2 (**B**,**C**), SMAD2 (**D**), pSMAD3 (**E**,**F**), SMAD3 (**G**), and YAP1 (**H**). The phosphorylated SMAD2 and SMAD3 proteins were normalized by both total SMAD2 (**B**) and SMAD3 (**E**), respectively, and β-actin (**C**,**F**). The levels of ‘total’ phosphorylated and unphosphorylated SMAD2 (**D**) and SMAD3 (**G**) were normalized by β-actin. YAP1 protein was normalized with β-actin (**H**). Three independent Western blots were used for protein quantification and statistical analysis (two-tailed Student’s *t* test with unpaired; *n* = 3 each group). *p*-values are shown in the figure. Numerical data from multiple individual samples are presented as scatter dot plots with error bars denoting the mean ± SD.

Cushion mesenchymal cells were treated with TGFβ2 in the absence and/or presence of verteporfin for 12 h. Western blot analyses were performed to quantify the changes in both phosphorylated (pSMAD2 and pSMAD3) and total SMAD2 and SMAD3 proteins. Total SMAD2 or SMAD3 proteins were used to normalize the expression of pSMAD2 or pSMAD3. YAP1 antibodies were used to measure the effect of verteporfin on Hippo pathway inhibition. The housekeeping β-actin protein was used to normalize the protein levels of total SMAD2, SMAD3, and YAP1. An equal number of cushion mesenchymal cells were used in all experiments (*n* = 3). Densitometric quantification of proteins from Western blots was performed. The data indicate higher levels of pSMAD2 (*n* = 3, *p* = 0.01) (Figure 5A–C) and pSMAD3 (*n* = 3, *p* = 0.02) (Figure 5A,E,F) following TGFβ2 treatment in cushion mesenchymal cells. The total SMAD2 or SMAD3 levels were not affected by TGFβ2 (Figure 5D,G). Verteporfin decreased the levels of YAP1 in the absence of TGFβ2 (*n* = 3, *p* = 0.003) or the presence of TGFβ2 (*n* = 3, *p* = 0.004) compared to control (Figure 5A,H). Densitometric data confirm that verteporfin significantly blocked the phosphorylation of pSMAD2 (Figure 5A–C) and pSMAD3 (Figure 5A,E,F). Finally, there was no significant effect of TGFβ2 and/or verteporfin on β-actin (Figure 5A). However, verteporfin treatment resulted in a significant downregulation of SMAD2 or SMAD3 in the absence or presence of TGFβ2 compared to the control or TGFβ2-treated cushion mesenchymal cells (Figure 5A,D,G). Collectively, the results indicate that verteporfin blocked YAP1 and both phosphorylated and unphosphorylated forms of SMAD2 and SMAD3.

## 4. Discussion

Although the TGFβ and Hippo pathways are involved in heart development and heart disease, the transcriptional regulatory mechanisms and how these pathways interact and regulate ECM remodeling remains to be fully understood. Several earlier studies reported that YAP1 and its transcriptional coactivator TAZ promote TGFβ signaling via retaining activated SMAD2/3 in the nucleus [8,26]. YAP1 remains inactive in the cytoplasm by the activated Hippo pathway. In the absence of Hippo activation, YAP1 stabilizes and subsequently translocate to the nucleus, where YAP1 acts as a transcriptional cofactor and induces the expression of several genes involved in differentiation and ECM remodeling during organ development [20]. This study is the first to provide evidence that YAP1 acts as a mediator of TGFβ2 function in cushion mesenchymal cells. In this study, cushion mesenchymal cells (tsA58-AVM cell line) are treated with TGFβ2 and/or verteporfin to determine if YAP1 inhibition interferes with SMAD3 activation and nuclear translocation. The cushion mesenchymal cell line used in this study is a very well-characterized and unique cell line to perform in vitro investigation to study cushion mesenchymal cell proliferation, apoptosis, ECM remodeling and cardiac cushion remodeling, differentiation, and maturation [28]. We chose TGFβ2 for the in vitro investigation in this study because a loss of TGFβ2 leads to major cardiovascular defects, including cardiac cushion remodeling defects [32,33,34]. Zhang et al. [22] studied the role of YAP1 in ‘endothelial cells’ in TGFβ1-induced endothelial to mesenchymal transition (EMT). Several studies have suggested that *Tgfb2* knockout but not *Tgfb1* knockout mice develop congenital heart defects [35,36]. Our study focuses on the role of TGFβ2 in embryonic cushion mesenchymal cells and not on cushion endothelial cells and investigates the function of YAP1 in TGFβ2-induced cushion mesenchymal cell remodeling and ECM expression. The AVM cell line used in this study was made from embryonic AV cushions. Unlike OFT cushions that have mesenchymal cells derived from both endocardium (via epithelial–mesenchymal transition, EMT) and neural crest cells, the AV cushion mesenchyme predominantly consists of endocardium-derived mesenchymal cells. Thus, our findings are mostly limited to the role of TGFβ and Hippo signaling in embryonic AV cushion mesenchymal cells. Overall, the findings presented in this study are novel and not confirmatory of a previously reported developmental analysis in mouse embryos [22].

Firstly, the immunofluorescence analysis indicates that TGFβ2 induces a robust nuclear accumulation of SMAD3 as well as YAP1 in cushion mesenchymal cells. A biochemical analysis confirmed that YAP1 inhibition effectively blocked the activation of SMAD2 and SMAD3 in cushion mesenchymal cells. The data also indicate that YAP1 inhibition results in decreased levels of both total SMAD2 and SMAD3 proteins suggesting that Hippo signaling acts as a negative mediator of canonical TGFβ–SMAD signaling in cushion mesenchymal cells. Together, the data suggest a role of the Hippo pathway in controlling TGFβ–SMAD signaling by regulating the transcription of *Smad2* or *Smad3* and/or post-translational (phosphorylation) changes and nuclear translocation of pSMAD2 and pSMAD3. Verteporfin has been shown to induce a dramatic reduction in YAP and TAZ that is associated not only with reduced SMAD2/3 nuclear accumulation, but also diminished SMAD2/3 levels after TGFβ stimulation in immortalized normal rat kidney interstitial fibroblasts (NRK49F) [37]. It is also known that YAP is a Smad7 partner that facilitates the recruitment of cytoplasmic YAP to activated TGFβR1 and enhances the inhibitory activity of Smad7 against TGFβ signaling in HaCaT keratinocytes and COS-7 cells [38]. Collectively, our results suggest that YAP1 can induce the expression and physically interact with activated SMAD2/3 within the cytoplasm and together they migrate to the nucleus in cushion mesenchymal cells. While in the nucleus, YAP1 independently or in conjunction with SMAD2/3 and in the presence of other transcriptional coactivators could regulate the transcription of key target genes in cushion cells. Some of the limitations of the current study is that this study has not addressed if there is any interaction between noncanonical TGFβ signaling (e.g., p38 MAPK and ERK1/2 MAPK) and the Hippo pathway and whether TGFβ signaling fails in the presence of the activated Hippo pathway [39]. Another limitation is that Western blot to confirm the findings of the mRNA levels of the ECM genes evaluated was not used. Because verteporfin significantly inhibits total SMAD3 levels, we were not able to use the pSMAD3/SMAD3 to monitor changes in pSMAD3. Instead, we used pSMAD3/β-actin and found that TGFβ2-induced pSMAD3 is significantly decreased in the presence of verteporfin. It is possible that verteporfin through a transcriptional and/or a post-transcriptional epigenetic mechanism can also have an impact on overall pSMAD3. This has not been investigated. Further investigation utilizing coimmunoprecipitation studies is required to determine if SMAD2/3 and YAP1 physically interact in cushion cells. It also remains unclear if TAZ and YAP1 respond redundantly to TGFβ2 in cushion mesenchymal cells. Finally, this is an in vitro investigation, and therefore future in vivo studies based on combinatorial genetics approaches are required to establish the functional interaction between TGFβ2 and Hippo signaling in ECM remodeling in cardiac development. Taken together, these results suggest that Hippo signaling could negatively modulate cell cushion mesenchymal cell behavior by interacting with the TGFβ pathway in heart valve development.

Secondly, the direct role of YAP1 in the ECM organization mediated by cushion mesenchymal cells in response to TGFβ2 and/or verteporfin is also investigated in the present study. TGFβ2 stimulated cushion mesenchymal cells in the reorganization of ECM in three-dimensional collagen gel contraction assays, and YAP1 inhibition blocked the TGFβ2-induced collagen contraction response. Because active Hippo signaling (Hippo-ON) normally deactivates YAP1 (YAP1-OFF), our data identify a negative role of the Hippo pathway in mediating the ability of cushion mesenchymal cells in reorganizing collagens in response to the exogenously added TGFβ2. The effect of TGFβ2 on collagen contraction seen in this study is comparable to the collagen contraction ability of mouse embryonic fibroblasts or cardiac fibroblasts by exogenously added TGFβ1, as reported by us [29,30,31,40]. Further investigation to elucidate the role of the TGFβ2 and Hippo pathways in vivo in cushion mesenchymal differentiation and maturation and ECM remodeling and reorganization processes will lead to a better understanding of heart development and heart disease.

Finally, given the importance of both the TGFβ and Hippo pathways in regulating genes involved in cardiac fibroblasts activation and ECM remodeling during development and cardiac disease, the expression of mesenchymal genes, such as *Acta2*, *Col1a1*, *Ccn2*, and *Ccn1*, was regulated by both TGFβ2 and/or Hippo signaling. TGFβ2 induces the expression of these ECM genes, whereas YAP1 inhibition blocks TGFβ2 regulation of these genes. These observations are consistent with the negative role of Hippo signaling in directly reorganizing collagen by exogenously added TGFβ2 in a three-dimensional gel contraction assay. Increased *Acta2, Ctgf* (*Ccn2*), and *Col1a1* expression is involved in tissue fibrosis [9,18] and tissue regeneration [41]. The loss of *Ccn1* or *Tgfb2* causes cardiac septal defects in humans and mice [33,34,42,43], suggesting a potential genetic and molecular interaction between the TGFβ2 and Hippo pathways. Mechanosensitive and profibrotic signaling pathways, such as YAP/TGFβ, are also implicated in multiple aspects of valvular heart disease and therefore considered potential targets for therapeutic interventions and prognostic biomarkers with the implications to improve the management of valvular heart disease [44]. Moreover, through the repurposing of a clinically used small-molecule inhibitor verteporfin, it is possible to alter TGFβ/SMAD signaling in vivo to investigate the role of TGFβ/Hippo pathway integration in heart development and heart disease.

In conclusion, Hippo signaling via YAP1 acts as a mediator of TGFβ2 function in cardiac cushion mesenchymal cells to regulate extracellular matrix remodeling. ECM organization is the hallmark of cushion mesenchymal differentiation and the maturation process during heart development. Thus, understanding the molecular regulation of TGFβ2-dependent ECM remodeling by YAP1 is an important step in elucidating the underlying mechanisms involved in the etiology of heart disease.

## Figures and Tables

**Table 1:** List of antibodies used to determine expression levels of different proteins.

Antibody	Company	Catalog No	Dilution
pSMAD2	Cell Signaling, Inc., Danvers MA, USA	3108S	1000
SMAD2	Cell Signaling, Inc., Danvers MA, USA	5339S	1000
pSMAD3	Cell Signaling, Inc., Danvers MA, USA	9520S	1000
SMAD3	Cell Signaling, Inc., Danvers MA, USA	9523S	1000
pSMAD1/5	Cell Signaling, Inc., Danvers MA, USA	9516S	1000
YAP1	abcam	ab205270	2000
HRP-anti-mice IgG	Cell Signaling, Inc., Danvers MA, USA	7076S	5000
HRP-anti-Rabbit IgG	Cell Signaling, Inc., Danvers MA, USA	7074S	5000
Goat anti-Rabbit, Cyanine3	Invitrogen, Waltham, MA, USA	A10520	1 µg/mL
β-actin	Sigma-Aldrich, St. Louis, MO, USA	A5441	10,000

**Table 2:** List of qPCR primers used to determine expression levels of different genes.

Sl. No	Target Gene	Biorad UniqueAssayID:
1	*Acta2*, Mouse	qMmuCID0006375
2	*Col1a1*, Mouse	qMmuCID0021007
3	*Ccn2*	qMmuCED0003632
4	*Ccn1*	qMmuCED0026152
5	*B2m*, Mouse	qMmuCID0040553

## Data Availability

The data presented in this study are available in this article.

## References

[1] Gittenberger-de Groot A.C., Bartelings M.M., DeRuiter M.C., Poelmann R.E. (2005). Basics of cardiac development for the understanding of congenital heart malformations. Pediatr. Res.

[2] Grewal N., DeRuiter M.C., Jongbloed M.R., Goumans M.J., Klautz R.J., Poelmann R.E., Gittenberger-de Groot A.C. (2014). Normal and abnormal development of the aortic wall and valve: Correlation with clinical entities. Neth. Heart J..

[3] Bosse K., Hans C.P., Zhao N., Koenig S.N., Huang N., Guggilam A., LaHaye S., Tao G., Lucchesi P.A., Lincoln J. (2013). Endothelial nitric oxide signaling regulates Notch1 in aortic valve disease. J. Mol. Cell Cardiol..

[4] Poelmann R.E., Gittenberger-de Groot A.C., Goerdajal C., Grewal N., De Bakker M.A.G., Richardson M.K. (2021). Ventricular Septation and Outflow Tract Development in Crocodilians Result in Two Aortas with Bicuspid Semilunar Valves. J. Cardiovasc. Dev. Dis..

[5] Burns T., Yang Y., Hiriart E., Wessels A. (2016). The Dorsal Mesenchymal Protrusion and the Pathogenesis of Atrioventricular Septal Defects. J. Cardiovasc. Dev. Dis..

[6] Calkoen E.E., Hazekamp M.G., Blom N.A., Elders B.B., Gittenberger-de Groot A.C., Haak M.C., Bartelings M.M., Roest A.A., Jongbloed M.R. (2016). Atrioventricular septal defect: From embryonic development to long-term follow-up. Int. J. Cardiol..

[7] Neeb Z., Lajiness J.D., Bolanis E., Conway S.J. (2013). Cardiac outflow tract anomalies. Wiley. Interdiscip. Rev. Dev. Biol..

[8] Attisano L., Wrana J.L. (2013). Signal integration in TGF-beta, WNT, and Hippo pathways. F1000Prime Rep..

[9] Tsai C.R., Martin J.F. (2022). Hippo signaling in cardiac fibroblasts during development, tissue repair, and fibrosis. Curr. Top. Dev. Biol..

[10] Piersma B., Bank R.A., Boersema M. (2015). Signaling in Fibrosis: TGF-beta, WNT, and YAP/TAZ Converge. Front. Med..

[11] Nallet-Staub F., Yin X., Gilbert C., Marsaud V., Ben Mimoun S., Javelaud D., Leof E.B., Mauviel A. (2015). Cell density sensing alters TGF-beta signaling in a cell-type-specific manner, independent from Hippo pathway activation. Dev. Cell.

[12] Frangogiannis N.G. (2017). The role of transforming growth factor (TGF)-beta in the infarcted myocardium. J. Thorac. Dis..

[13] Hanna A., Humeres C., Frangogiannis N.G. (2021). The role of Smad signaling cascades in cardiac fibrosis. Cell Signal..

[14] Lindsay M.E., Schepers D., Bolar N.A., Doyle J.J., Gallo E., Fert-Bober J., Kempers M.J., Fishman E.K., Chen Y., Myers L. (2012). Loss-of-function mutations in TGFB2 cause a syndromic presentation of thoracic aortic aneurysm. Nat. Genet..

[15] Boileau C., Guo D.C., Hanna N., Regalado E.S., Detaint D., Gong L., Varret M., Prakash S.K., Li A.H., d’Indy H. (2012). TGFB2 mutations cause familial thoracic aortic aneurysms and dissections associated with mild systemic features of Marfan syndrome. Nat. Genet..

[16] Heldin C.H., Moustakas A. (2016). Signaling Receptors for TGF-beta Family Members. Cold Spring Harb. Perspect. Biol..

[17] Sun C., Zhang H., Liu X. (2021). Emerging role of CCN family proteins in fibrosis. J. Cell Physiol..

[18] Mia M.M., Singh M.K. (2022). New Insights into Hippo/YAP Signaling in Fibrotic Diseases. Cells.

[19] Mia M.M., Singh M.K. (2019). The Hippo Signaling Pathway in Cardiac Development and Diseases. Front. Cell Dev. Biol..

[20] Ma S., Meng Z., Chen R., Guan K.L. (2019). The Hippo Pathway: Biology and Pathophysiology. Annu. Rev. Biochem..

[21] Misra J.R., Irvine K.D. (2018). The Hippo Signaling Network and Its Biological Functions. Annu. Rev. Genet..

[22] Zhang H., von Gise A., Liu Q., Hu T., Tian X., He L., Pu W., Huang X., He L., Cai C.L. (2014). Yap1 is required for endothelial to mesenchymal transition of the atrioventricular cushion. J. Biol. Chem..

[23] Pobbati A.V., Hong W. (2013). Emerging roles of TEAD transcription factors and its coactivators in cancers. Cancer Biol. Ther..

[24] Liu-Chittenden Y., Huang B., Shim J.S., Chen Q., Lee S.J., Anders R.A., Liu J.O., Pan D. (2012). Genetic and pharmacological disruption of the TEAD-YAP complex suppresses the oncogenic activity of YAP. Genes Dev..

[25] Lockhart M., Wirrig E., Phelps A., Wessels A. (2011). Extracellular matrix and heart development. Birth Defects Res. A Clin. Mol. Teratol..

[26] Labibi B., Bashkurov M., Wrana J.L., Attisano L. (2020). Modeling the Control of TGF-beta/Smad Nuclear Accumulation by the Hippo Pathway Effectors, Taz/Yap. iScience.

[27] Gori I., George R., Purkiss A.G., Strohbuecker S., Randall R.A., Ogrodowicz R., Carmignac V., Faivre L., Joshi D., Kjaer S. (2021). Mutations in SKI in Shprintzen-Goldberg syndrome lead to attenuated TGF-beta responses through SKI stabilization. Elife.

[28] Peng Y., Song L., Li D., Kesterson R., Wang J., Wang L., Rokosh G., Wu B., Wang Q., Jiao K. (2016). Sema6D acts downstream of bone morphogenetic protein signalling to promote atrioventricular cushion development in mice. Cardiovasc. Res..

[29] Carver W., Fix E., Fix C., Fan D., Chakrabarti M., Azhar M. (2021). Effects of emodin, a plant-derived anthraquinone, on TGF-beta1-induced cardiac fibroblast activation and function. J. Cell Physiol..

[30] Haskett D., Doyle J.J., Gard C., Chen H., Ball C., Estabrook M.A., Encinas A.C., Dietz H.C., Utzinger U., Vande Geest J.P. (2012). Altered tissue behavior of a non-aneurysmal descending thoracic aorta in the mouse model of Marfan syndrome. Cell Tissue Res..

[31] Fix C., Carver-Molina A., Chakrabarti M., Azhar M., Carver W. (2019). Effects of the isothiocyanate sulforaphane on TGF-beta1-induced rat cardiac fibroblast activation and extracellular matrix interactions. J. Cell Physiol..

[32] Bartram U., Molin D.G., Wisse L.J., Mohamad A., Sanford L.P., Doetschman T., Speer C.P., Poelmann R.E., Gittenberger-de G.A. (2001). Double-outlet right ventricle and overriding tricuspid valve reflect disturbances of looping, myocardialization, endocardial cushion differentiation, and apoptosis in *Tgfb2* knockout mice. Circulation.

[33] Ishtiaq Ahmed A.S., Bose G.C., Huang L., Azhar M. (2014). Generation of mice carrying a knockout-first and conditional-ready allele of transforming growth factor beta2 gene. Genesis.

[34] Bhattacharya A., Al-Sammarraie N., Gebere M.G., Johnson J., Eberth J.F., Azhar M. (2021). Myocardial TGFbeta2 Is Required for Atrioventricular Cushion Remodeling and Myocardial Development. J. Cardiovasc. Dev. Dis..

[35] Azhar M., Runyan R.B., Gard C., Sanford L.P., Miller M.L., Andringa A., Pawlowski S., Rajan S., Doetschman T. (2009). Ligand-specific function of transforming growth factor beta in epithelial-mesenchymal transition in heart development. Dev. Dyn..

[36] Azhar M., Yin M., Bommireddy R., Duffy J.J., Yang J., Pawlowski S.A., Boivin G.P., Engle S.J., Sanford L.P., Grisham C. (2009). Generation of mice with a conditional allele for transforming growth factor beta 1 gene. Genesis.

[37] Szeto S.G., Narimatsu M., Lu M., He X., Sidiqi A.M., Tolosa M.F., Chan L., De Freitas K., Bialik J.F., Majumder S. (2016). YAP/TAZ Are Mechanoregulators of TGF-beta-Smad Signaling and Renal Fibrogenesis. J. Am. Soc. Nephrol..

[38] Ferrigno O., Lallemand F., Verrecchia F., L’Hoste S., Camonis J., Atfi A., Mauviel A. (2002). Yes-associated protein (YAP65) interacts with Smad7 and potentiates its inhibitory activity against TGF-beta/Smad signaling. Oncogene.

[39] Huang D., Li X., Sun L., Huang P., Ying H., Wang H., Wu J., Song H. (2016). Regulation of Hippo signalling by p38 signalling. J. Mol. Cell Biol..

[40] Chakrabarti M., Al-Sammarraie N., Gebere M.G., Bhattacharya A., Chopra S., Johnson J., Pena E.A., Eberth J.F., Poelmann R.E., Gittenberger-de Groot A.C. (2020). Transforming Growth Factor Beta3 is Required for Cardiovascular Development. J. Cardiovasc. Dev. Dis..

[41] Wang J., Liu S., Heallen T., Martin J.F. (2018). The Hippo pathway in the heart: Pivotal roles in development, disease, and regeneration. Nat. Rev. Cardiol..

[42] Mo F.E., Lau L.F. (2006). The matricellular protein CCN1 is essential for cardiac development. Circ. Res..

[43] Perrot A., Schmitt K.R., Roth E.M., Stiller B., Posch M.G., Browne E.N., Timmann C., Horstmann R.D., Berger F., Ozcelik C. (2015). CCN1 mutation is associated with atrial septal defect. Pediatr. Cardiol..

[44] Dayawansa N.H., Baratchi S., Peter K. (2022). Uncoupling the Vicious Cycle of Mechanical Stress and Inflammation in Calcific Aortic Valve Disease. Front. Cardiovasc. Med..

